# Genetic parameters for milk fatty acid composition of Holstein in Korea

**DOI:** 10.5713/ajas.19.0820

**Published:** 2020-02-25

**Authors:** Chan Hyuk Park, Umanthi Ranaraja, Chang Gwon Dang, Jong Joo Kim, Chang Hee Do

**Affiliations:** 1 Division of Animal and Dairy Science, Chungnam National University, Daejeon 34134, Korea; 2 National Institute of Animal Science, RDA, Cheonan 31000, Korea; 3 School of Biotechnology, Yeungnam University, Gyeongsan 38541, Korea

**Keywords:** Heritability, Parity, Energy Balance, Lactation

## Abstract

**Objective:**

Milk fatty acid (FA) is a main nutritional component that markedly effects human health. Intentional modification of the FA profile has the potential to improve milk quality. This study aimed at the factors affecting elevated FA levels and the estimation of the genetic parameters for milk FAs in the Korean Holstein population.

**Methods:**

Total 885,249 repeated test-day milk records including, milk yield, saturated fatty acids (SFA), polyunsaturated fatty acids (PUFA), monounsaturated fatty acids (MUFA), total unsaturated fatty acids (TUFA), fat and protein percentages were analyzed using CombiFoss FT+ system (Foss Analytical A/S, Denmark). Genetic parameters were estimated by the restricted maximum likelihood procedure based on the repeatability model using the Wombat program.

**Results:**

The FA profile varies along with the lactation and the energy balance (EB). With the negative EB in early lactation, mobilization of body fat reserves elevates the desirable FA levels. As a result of that, milk quality is increased by means of nutritionally and usability aspects during the early lactation. Moreover, heritability estimates for SFA, MUFA, PUFA, TUFA were 0.33, 0.42, 0.37, 0.41 respectively. According to the parity wise heritability analysis, first parity cows had relatively lower heritability for SFAs (0.19) than later parities (0.28).

**Conclusion:**

Genetic parameters indicated that FAs were under stronger genetic control. Therefore, we suggest implementing animal breeding programs towards improving the milk FA profile.

## INTRODUCTION

The objective of this study was to evaluate the phenotypic and genetic variability of fatty acid (FA) groups and identify the factors that influence changes in the FA profile of the Korean Holstein population. The milk fatty acids are derived from four major pathways. Directly from the diet, de novo synthesis in the mammary gland, formation in the rumen by biohydrogenation or bacterial degradation, and release from body fat stores [1,2].

Practical efforts to improve the milk FA profile to increase the benefits to the consumers are driven by two reasons based on nutritional and usability aspects. A concern for dairy consumers is that the nutritional approach leads to a lower proportion of unsaturated fatty acids (UFAs) and a higher proportion of saturated fatty acids (SFAs). Consequently, there can be various deleterious effects on human health like cardiovascular disease risk, elevated blood pressure, insulin resistance, and hyperlipidemia, particularly of low-density lipoprotein cholesterol [3,4].

From a usability point of view, textural properties of milk and butter are known to be affected by FA composition. Higher proportions of UFA are desired due to more spreadable and softer butter, with less adhesive consistency of milk and butter. However, there are some issues related to high UFA content in milk fat, including its lower stability and the accompanying phenomena such as oxidation and possible sensory changes [5].

Making Intentional modifications to the FA profile requires a thorough knowledge of the different factors that effect on milk fat composition and the extent to which these relevant factors are involved in influencing the FA profile. According to the previous literature, factors affecting the FA profile of milk are breed, cow’s individuality, milk yield, lactation (parity and stage), feed composition, management and metabolic factors [6]. But these factors continue to be studied because of their combined effects with a wide range of variations. Several studies reported the effect of breed and dieton milk fat composition [7–9]. Besides that, changes in energy status over lactation have an impact on fatty acid profile [10]. During the early lactation, the occurrence of a negative energy balance (NEB) is common in dairy cows. The deficiency of nutrients and energy is compensated by mobilization of body reserves, mainly adipose tissue associated with the release of FA.Along with that, some studies have investigated genetic effects on milk FA profiles [11,12] and the evolution of heritabilities and genetic correlations of FA contents across a lactation [13]. These studies were generally based on a limited number of records.

The focus of this study was to evaluate the effect of parity, lactation stage, and energy balance on the contents of fatty acids and estimate their genetic parameters according to the parity and collectively. Selection for improved FA profiles would be feasible only if there is sufficient genetic variation in FA composition.

## MATERIALS AND METHODS

### Data

Test-day records of milk composition were collected from 2012 to 2018 by the Korea Animal Improvement Association (KAIA). The test day milk records included milk yield, FA composition, (monounsaturated fatty acids [MUFA], polyunsaturated fatty acids [PUFA], SFA, total unsaturated fatty acids [TUFA]) fat and protein percentages of cows that were 1 to 305 days in milk at sampling. The cows were milked twice daily at morning (05:00 h) and afternoon (16:00 h). Milk composition was analyzed weekly based on samples collected from 2 consecutive milkings. Test-day milk samples were analyzed by FTIR spectroscopy using the CombiFossFT+ system (Foss Analytical A/S, Hillerod, Denmark).

The season of calving was defined as summer (May to October) and winter (November to April). The age at first calving ranged from ≤23 months, 24 to 25 months, 26 to 28 months and ≥29 respectively. The original data set consisted of 885,249 test-day records.

### Statistical analysis

SAS 9.2 package (SAS Institute Inc. Cary, NC, USA) was used to analyze descriptive statistics of all parameters. Genetic parameters, including genetic (co)variance components, were estimated by the restricted maximum likelihood procedure based on a repeatability model using the Wombat program.

The linear model for all parities and individual parities were illustrated as follows (Model 1 and Model 2):

Model 1 $${\text{Y}}_{\text{ijklm}}=\mu +\text{DIM}+{\text{Age}}_{\text{i}}+{\text{Season}}_{\text{j}}+{\text{ampm}}_{\text{k}}+{\text{parity}}_{\text{l}}+{\text{a}}_{\text{m}}+{\text{p}}_{\text{m}}+{\text{e}}_{\text{ijklm}}$$

Model 2 $${\text{Y}}_{\text{ijklm}}=\mu +\text{DIM}+{\text{Age}}_{\text{i}}+{\text{Season}}_{\text{j}}+{\text{ampm}}_{\text{k}}+{\text{a}}_{\text{m}}+{\text{p}}_{\text{m}}+{\text{e}}_{\text{ijkm}}$$

Where, Y_ijklm_ is the SFA, MUFA, PUFA, TUFA observation; μ is the overall mean; DIM is the covariate describing the effect of days in milk; Age_i_ is the fixed effect of calving age I; Season_j_ is the fixed effect of calving season j; ampm_k_ is the fixed effect of milk collecting time k; parity_l_ is the fixed effect of parity l; a_m_ is the additive genetic effect of cow m; p_m_ is the permanent environmental effect of cow m; e is the random residual effect associated with each record.

Heritability was calculated using this equation:

$$h^{2}=\frac{\sigma_{\text{A}}^{2}}{\sigma_{\text{P}}^{2}}$$

Where *h*^2^ is heritability, $\sigma_{\text{A}}^{2}$ is additive genetic variance and $\sigma_{\text{P}}^{2}$ is phenotypic variance.

This EB equation was based on parity, lactation week, and milk composition volume as follows [14,15].

Equation

$$\begin{array}{ll} \text{eEB } & =217.8-\text{wk}2\times 31.9-\text{wk}3\times 20.6-\text{wk}4\times 15.6\\ & -\text{wk}5\times 11.5-\text{wk}6\times 8.0-\text{wk}7\times 10.6-\text{wk}8\times 7.2\\ & -\text{wk}9\times 5.3-\text{wk}10\times 4.0-\text{wk}11\times 2.7-\text{wk}12\times 0\\ & -\text{par}1\times 34.9-\text{par}2\times 7.2-\text{par}3\times 6.7-\text{par}4\times 0\\ & -\text{milk}\times 2.11-\text{prot}\times 15.36-\text{FP}\times 49.24\;(\text{MJ nel}/\text{d}) \end{array}$$

Where; wk2, wk3 . . . to wk12 = lactation wk2 to 12; par1 – par4 = parity categories 1 to 4; milk = milk yield (kg/d); prot = % milk protein; FP = ratio of % fat to % protein in milk.

## RESULTS AND DISCUSSION

A total of 885,249 milk samples was evaluated for SFA, MUFA, PUFA, TUFA at KAIA. Mean SFA content was 2.43 g/dL of milk with the range of 0.05 g/dL of milk to 14.2 g/dL of milk while the mean TUFA content was 1.29 g/dL of milk with the range of 0.01 g/dL of milk to 8.03 g/dL of milk. The average milk yield was 17.34 kg/d while mean fat and protein percentages were 3.83 and 3.24 respectively (Table 1).

**Figure 1:**
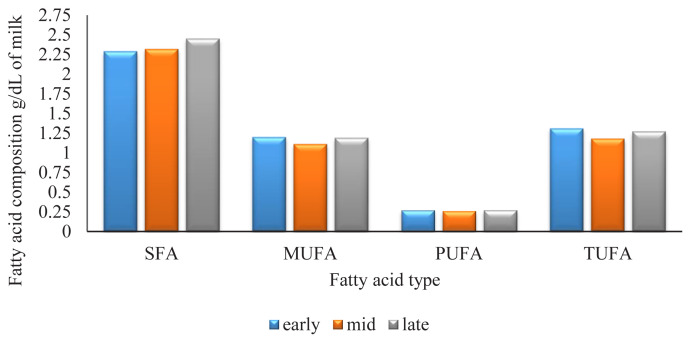
Fatty acid composition in different lactation stages. SFA, saturated fatty acid; MUFA, monounsaturated fatty acid; PUFA, polyunsaturated fatty acid; TUFA, total unsaturated fatty acid; DIM, days in milk. Early lactation, 1 to 100 DIM; midlactation, 101 to 200 DIM; late lactation, 201 to 305 DIM.

In this study stage of lactation was considered as a class variable with 3 levels early lactation (1 to 100 d), mid-lactation (101 to 200 d), and late lactation (200 to 305 d). SFA increased over lacation. SFA content at early lactation was 2.29 g/dL of milk, 2.32 g/dL of milk at mid-lactation and 2.45 g/dL of milk at late lactation. The patterns for MUFA, and TUFA were similar to one another and showed a minimum at mid-lactation, PUFA content was not observed a significant change over lactation (Figure 1).

Lactation stage, along with energy balance of dairy cows, has an impact on the FA profile of cow’s milk. Changes in milk FA composition during lactation, predominantly at the beginning of lactation, originate from alterations in the pathways of FA derivation, the diet, de novo synthesis in mammary glands, ruminal biohydrogenation and body fat mobilization.

During lactation, the cycles of lipolysis and lipogenesis in body stores are altering to meet her energy requirements for milk secretion. The increased energy demands of fetal development and milk secretion are mainly evident in the transition period of lactation. Therefore, cows, like other lactating animals, often enter a NEB at the start of lactation [16].

**Figure 2:**
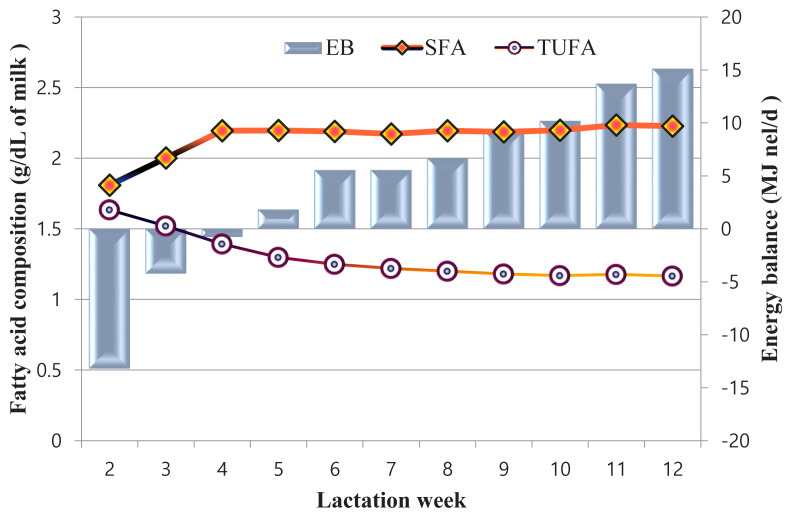
The relationship between milk SFA and TUFA concentration with energy balance along with lactation week. SFA, saturated fatty acid; TUFA, total unsaturated fatty acid; EB, energy balance.

Figure 2 shows the relationship between mean EB and fatty acid composition. In the 2nd week of lactation, EB was −13.18 MJ nel/d and it increased up to 63.40 MJ nel/d by the 12th week of lactation. During the phase of negative EB a low level of SFA content was observed which increased with EB. TUFA content was high in the second week of lactation and it slightly decreased and even out with the progression of lactation (Figure 2).

Early lactation is the most challenging period in terms of energy status and herd management. High utilization of energy reserves during this period is reflected in milk fat content mainly in the FA composition and mutual ratios between individual FA groups. The general pattern can be described as high uptake of long-chain FAs by the mammary gland affecting the de novo synthesis of FAs. Therefore, SFAs are at their lowest proportion in the second week, with increasing amounts until 12 weeks as the energy balance improves [17]. These findings agreed with our recent results.

Estimates of genetic parameters for fatty acids proportions in milk fat are required to make genetic selection decisions. In this study, heritability estimates for SFA, MUFA, PUFA, TUFA were 0.33, 0.42, 0.37, 0.41 respectively (Table 2). High heritabilities indicated that de novo synthesized FA were under stronger genetic control and that selection of animals with improved FA profiles would be feasible due to genetic variation in FA composition.

Stoop et al [12] reported estimates of heritabilities in the range of 0.09 to 0.54 for individual C4:0 to C18:3 fatty acid percentages in milk fat in the first lactation Dutch Holsteins. Bastin et al [18] also reported a higher range of heritability estimates (0.18 to 0.44) for 12 individual fatty acids (g/dL of milk) in the milk of first lactation Holstein cows in Belgium. Karijord et al [19] reported phenotypic and genetic correlation estimates among fatty acids in bovine milk fat using a sire model and found large positive phenotypic and genetic correlation estimates among short-chain fatty acids. In this study all genetic and phenotypic correlations were positive.

There is conflicting evidence in the literature about the effect of parity on the fatty acid composition of bovine milk. According to the parity wise heritability analysis, first parity cows showed a lower heritability in SFA than higher parities (0.19). There was no significant difference between heritabilities in other parities (Table 3). In the Canadian Holstein population, it was reported that the fatty acid composition of milk did not differ across parities [20]. In contrast, parity has been reported a significant effect on the contents of fatty acids (conjugated linoleic acid) in milk of US Holstein and Brown Swiss cows [21]. First-parity cows had a relatively high amount of desirable fatty acids and lower proportions of less desirable fatty acids compared with later parity cows, suggesting that the rate of unsaturation of saturated fatty acid was lower for first-parity cows as compared with cows in later parities [22]. As first parity cows are still growing as compared to later parity cows, this might have implications for the milk fatty acid synthesis in dairy cows. Miller et al [23] demonstrated that the mammary gland of first parity cows was metabolically less active than later parity cows and noted a lower expression of fatty acid synthase in the mammary gland of the first parity cows in early lactation.

Mentioned findings and relationships confirm the impor tance of maintaining the fatty acid composition in Korean Holstein cows in relation to the economic effectiveness of dairy milk with high quality. Our study showed that the lactation stage, energy balance and parity significantly contribute to variation in milk fat composition. So, this can be used in future breeding programs to improve the FA profile of milk fat by increasing UFA concentrations and decreasing SFA concentrations.

## CONCLUSION

According to our findings, the milk FA profile in early lactation is healthier for the consumer. The FA composition of milk depends on the parity, stage of lactation, and energy balance. Overall high heritability estimates of FAs suggested that milk fat composition can be changed by genetic selection. The test day records from commercial herds are an effective tool for genetic selection directed towards improving milk quality. Moreover, we conclude that milk and other dairy products can be labeled by parity and the stage of lactation at the market level.

## Figures and Tables

**Table 1:** Descriptive statistics for the traits including productive traits

Traits	No of records	Mean	SD	Minimum	Maximum
Protein (%)	885,249	3.24	0.32	0.4	12.16
Fat (%)	885,249	3.83	0.97	0.11	18.89
SFA (g/dL of milk)	885,249	2.43	0.68	0.05	14.2
MUFA (g/dL of milk)	885,249	1.21	0.27	0.02	6.05
PUFA (g/dL of milk)	885,249	0.27	0.04	0.01	0.86
TUFA (g/dL of milk)	885,249	1.29	0.41	0.01	8.03
Milk yield (kg/d)	885,249	17.34	4.74	11.8	98

SD, standard deviation; SFA, saturated fatty acid; MUFA, monounsaturated fatty acid; PUFA, polyunsaturated fatty acid; TUFA, total unsaturated fatty acid.

**Table 2:** Heritabilities (in bold in the diagonal) and genetic correlations (above the diagonal) and phenotypic correlations (below diagonal)

Items	SFA	MUFA	PUFA	TUFA
SFA	**0.33**	0.117	0.015	0.093
MUFA	0.091	**0.42**	0.171	0.051
PUFA	0.010	0.129	**0.37**	0.128
TUFA	0.073	0.056	0.097	**0.41**

SFA, saturated fatty acid; MUFA, monounsaturated fatty acid; PUFA, polyunsaturated fatty acid; TUFA, total unsaturated fatty acid.

**Table 3:** Heritabilities (in bold in the diagonal) and genetic correlations (above the diagonal) and phenotypic correlations (below diagonal) according to the parity

Items	SFA	MUFA	PUFA	TUFA
Parity1				
SFA	**0.1941**	0.0056	0.2873	0.0045
MUFA	0.0034	**0.3992**	0.2613	0.5121
PUFA	0.1936	0.2090	**0.44487**	0.4102
TUFA	0.0027	0.0021	0.1837	**0.4067**
Parity2				
SFA	**0.2860**	0.0043	0.2299	0.0035
MUFA	0.0034	**0.3996**	0.2154	0.0021
PUFA	0.1962	0.2125	**0.4552**	0.1878
TUFA	0.0028	0.0020	0.1917	**0.4080**
Parity3				
SFA	**0.2883**	0.0039	0.2083	0.0031
MUFA	0.0030	**0.3899**	0.1923	0.0020
PUFA	0.1745	0.1822	**0.4637**	0.1609
TUFA	0.0024	0.0019	0.1601	**0.3965**
Parity4				
SFA	**0.2849**	0.0021	0.1590	0.0017
MUFA	0.0016	**0.3811**	0.1400	0.0012
PUFA	0.1326	0.1311	**0.4696**	0.1119
TUFA	0.0013	0.0011	0.1109	**0.3909**

SFA, saturated fatty acid; MUFA, monounsaturated fatty acid; PUFA, polyunsaturated fatty acid; TUFA, total unsaturated fatty acid.
